# Is muscular strength compromised by overnight fasting or food ingestion in hospital settings?

**DOI:** 10.31744/einstein_journal/2019CE5318

**Published:** 2019-11-13

**Authors:** Renata da Rocha Muniz Rodrigues, Joana Maia Brandão, Danilo Cosme Klein Gomes, Rafael Lavourinha Pinto

**Affiliations:** 1 Universidade do Estado do Rio de Janeiro, Rio de Janeiro, RJ, Brazil.

Dear Editor,

The article “Effects of overnight fasting on handgrip strength in inpatients”^1^ is relevant and has practical application in the hospital environment. Nevertheless, a few methodological issues need to be clarified:

(1) There is no reference measure of fasting time, considering the nighttime fasting is physiological, common to all patients. What the authors really investigated was the percentage of food ingestion offered on the previous day and its association with handgrip strength (HGS). There is no way to investigate the causal relation between fasting and HGS, since there is no information as to time of duration of the fast by the study participants. Additionally, it is known that in a hospital routine, a meal is offered after dinner, and this meal is not even mentioned by the authors.^2^

(2) The values described for fasting HGS, after eating breakfast, lunch, and accumulated ingestions are so similar among themselves, that the doubt remains if the difference among them is, in fact, relevant, even after presenting with significant p values. Furthermore, the authors did not present reference values for HGS in individuals within the age range encompassed by the study, impeding any type of comparison with the population.^3^

As to the presentation of the results, there is an exaggerated use of graphs and tables, containing information already covered in the text, without adding relevant data. There is a need for tighter controls in selection, *e.g.,* of figures 1, 2, and 3, which do not require a graphic presentation, since they are data easily described in the text. Yet, figure 4 makes comprehension more difficult, for it does not clearly describe how the groups were defined. There is also the redundancy in presenting the p value and of the odds ratio for the same analysis.

The authors found relevant data, but this was not appreciated. The core issue of the study should be the influence of calorie ingestion on the day before on HGS, characterizing a worsening nutritional status. This point should be better explored, discussing the importance of improving the quantity ingested of the prescribed diet to inpatients, minimizing the reduction in HGS and the consequent nutritional modifications.

Considering the points mentioned here, we conclude that the authors erroneously analyzed the results, generating imprecise interpretations.
