# Supplementary material for: Is muscular strength compromised by overnight fasting or food ingestion in hospital settings?
Source: Einstein (Sao Paulo). 2019 Nov 13;17(4):eCE5318. doi: 10.31744/einstein_journal/2019CE5318 (PMC6855697; doi:10.31744/einstein_journal/2019CE5318)
Supplement: Translation

# Translation: A força muscular é comprometida pelo jejum noturno ou pelo percentual de ingestão da dieta hospitalar?

**Authors:** Renata da Rocha Muniz Rodrigues, Joana Maia Brandão, Danilo Cosme Klein Gomes, Rafael Lavourinha Pinto

**Affiliations:** 1 Universidade do Estado do Rio de Janeiro, Rio de Janeiro, RJ, Brasil.

Caro Editor,

O artigo “Efeitos do jejum noturno sobre a força muscular em pacientes internados”^1^é relevante e de aplicação prática no ambiente hospitalar. Porém, algumas questões metodológicas merecem ser esclarecidas:

(1) Não há referência da mensuração do tempo de jejum, considerando que o jejum noturno é algo fisiológico, comum a todos os pacientes. O que os autores na verdade investigaram foi o percentual de ingestão da alimentação ofertada no dia anterior e sua associação com a força de preensão palmar (FPP). Não há como investigar a relação causal entre jejum e FPP, dado que não há informação do tempo de permanência em jejum pelos participantes do estudo. Além disso, sabe-se que, na rotina hospitalar, existe a oferta da ceia após o jantar, refeição essa que sequer foi citada pelos autores.^2^

(2) Os valores descritos de FPP em jejum, após a ingestão do desjejum, do almoço e acumulado são tão similares entre si, que fica a dúvida se a diferença entre eles é, de fato, relevante, mesmo apresentando valores de p significativos. Além disso, os autores não apresentaram valores de referência para FPP em indivíduos na faixa etária compreendida pelo estudo, impossibilitando algum tipo de comparação com a população.^3^

Quanto à apresentação dos resultados, há uso exagerado de gráficos e tabelas, que contêm informações já contempladas no texto, sem acrescentar dados relevantes. Seria necessário maior rigor na seleção, por exemplo, das figuras 1, 2 e 3, que não requerem apresentação gráfica, pois os dados são facilmente descritos no texto. Ainda, a figura 4 torna confusa a compreensão, pois não descreve com clareza como os grupos foram definidos. Existe também a redundância em apresentar o valor de p e as *odds ratio* para a mesma análise.

Os autores encontraram dados relevantes, mas isso não foi valorizado. O cerne do trabalho deveria ser a influência da ingestão calórica do dia anterior na FPP, caracterizando piora do estado nutricional. Este ponto deveria ser mais explorado, discutindo a importância de melhorar a quantidade ingerida da dieta prescrita aos pacientes internados, minimizando a redução da FPP e as consequentes alterações nutricionais.

Considerando os pontos aqui mencionados, conclui-se que os autores analisaram de forma equivocada os resultados, gerando interpretações imprecisas.
